# Association Mapping of Disease Resistance Traits in Rainbow Trout Using Restriction Site Associated DNA Sequencing

**DOI:** 10.1534/g3.114.014621

**Published:** 2014-10-28

**Authors:** Nathan R. Campbell, Scott E. LaPatra, Ken Overturf, Richard Towner, Shawn R. Narum

**Affiliations:** * Columbia River Inter-Tribal Fish Commission, Hagerman, Idaho 83332; † Clear Springs Foods Inc., Research Division, Buhl, Idaho 83316; ‡ USDA-ARS, Hagerman, Idaho 83332; § GeneTec Consulting, Payette, Idaho 83661

**Keywords:** genotyping by sequencing, infectious hematopoietic necrosis virus, cold water disease, *Flavobacterium psychrophilum*, genetics of immunity

## Abstract

Recent advances in genotyping-by-sequencing have enabled genome-wide association studies in nonmodel species including those in aquaculture programs. As with other aquaculture species, rainbow trout and steelhead (*Oncorhynchus mykiss*) are susceptible to disease and outbreaks can lead to significant losses. Fish culturists have therefore been pursuing strategies to prevent losses to common pathogens such as *Flavobacterium psychrophilum* (the etiological agent for bacterial cold water disease [CWD]) and infectious hematopoietic necrosis virus (IHNV) by adjusting feed formulations, vaccine development, and selective breeding. However, discovery of genetic markers linked to disease resistance offers the potential to use marker-assisted selection to increase resistance and reduce outbreaks. For this study we sampled juvenile fish from 40 families from 2-yr classes that either survived or died after controlled exposure to either CWD or IHNV. Restriction site−associated DNA sequencing produced 4661 polymorphic single-nucleotide polymorphism loci after strict filtering. Genotypes from individual survivors and mortalities were then used to test for association between disease resistance and genotype at each locus using the program TASSEL. After we accounted for kinship and stratification of the samples, tests revealed 12 single-nucleotide polymorphism markers that were highly associated with resistance to CWD and 19 markers associated with resistance to IHNV. These markers are candidates for further investigation and are expected to be useful for marker assisted selection in future broodstock selection for various aquaculture programs.

The practice of marker-assisted selection (MAS) uses sets of genetic markers linked to desirable traits to guide breeding decisions for production of agricultural species. In fact, MAS has proven to be a useful tool for the development of agricultural animal and plant strains with dramatically improved trait characteristics (*i.e.*, Han *et al.* 1997; Serraj *et al.* 2005; Singh *et al.* 2001). Global aquaculture of rainbow trout and steelhead (*Oncorhynchus mykiss*) is extensive for commercial, conservation, and harvest purposes, but the use of MAS to improve traits such as disease resistance is currently lacking. For MAS to become useful for rainbow trout trait selection, closely associated genetic markers must first be identified from a dense panel of markers throughout the genome. To this point, association and quantitative trait loci (QTL) mapping approaches in this species have relied on relatively small numbers of genetic markers. These approaches have either failed to identify highly associated loci (Overturf *et al.* 2010) or identified significant associations within very large blocks of linkage disequilibrium (LD; Rodriguez *et al.* 2004; Barroso *et al.* 2008). However, recent advancements in high-throughput sequencing technology have made genotyping-by-sequencing methods possible, bypassing the need for expensive a-priori marker identification (*i.e.*, Davey *et al.* 2011; Hess *et al.* 2012; Keller *et al.* 2013; Narum *et al.* 2013). Methods such as restriction site−associated sequencing (RAD) have enabled genome-wide association studies (GWAS) with large numbers of single-nucleotide polymorphisms (SNPs) for species with limited genomic resources (*e.g.*, Hecht *et al.* 2012).

As with other aquaculture species, *O. mykiss* are susceptible to disease and outbreaks (especially among juveniles) can result in significant losses (Lapatra 1998; LaPatra *et al.* 2001a). As such, precautions are taken to ameliorate disease outbreaks through better management practices such as using disinfecting foot baths, iodine treatment of eggs, and limiting reused water. However, disease outbreaks can still occur through various sources, such as transmission from wild sources or asymptomatic infection within the hatchery population. Other approaches such as immune boosting feed formulations (*i.e.*, Navarre and Halver 1989; Brunt *et al.* 2007) and vaccination against common pathogens also have been explored (Alvarez *et al.* 2008; LaFrentz and LaPatra 2003; Lapatra *et al.* 2001b), although these approaches also increase production costs.

Another approach to develop disease-resistant rainbow trout strains is through selective breeding. Resistance to disease is a difficult trait to phenotype, and methods for quantification of disease resistance have used offspring mortality rates after pathogen exposure to measure parent breeding potential (*e.g.*, Henryon *et al.* 2005). Selection of broodstock fish is then based on mortality rates rather than phenotype for selective breeding purposes. Over generations, gene variants within the cultivated fish strain that are beneficial for disease resistance are enhanced whereas those associated with disease manifestation are diminished. This method has been used to create rainbow trout strains with improved resistance to a few common pathogens. However, selected strains remain susceptible to other diseases, and the selection process may negatively affect favorable production traits such as growth rate (Henryon *et al.* 2002).

In this study RAD sequencing was used to identify genetic markers within a population of cultured rainbow trout and test for significant associations with disease resistance. A set of disease resistance−associated genetic markers was then analyzed further to assess their ability to discriminate susceptible from resistant fish segregating within the population.

## Materials and Methods

### Ethics statement

This study used rainbow trout fin clips collected after controlled exposure to the fish pathogens *F. psychrophilum* and IHNV as part of a selective breeding program at the Clear Springs Foods Inc. research facility. As farm animals used in a commercial breeding program, these fish are exempted from regulation under the U.S. Animal Welfare Act and therefore not subject to oversight by an Institutional Animal Care and Use Committee or other such ethics committee. This exemption is defined in U.S. Code title 7, chapter 54, section 2132g. However, experimentation and handling was conducted in accordance to U.S. government principals for the use and care of vertebrate animals used in testing, research, and training, which includes provisions to minimize animal suffering. Specific measures for amelioration of animal suffering during the fish pathogen testing (described in detail in *Disease challenge and sample collection*) included minimization of handling, maintenance of optimal water temperature, and oxygen saturation, and the fish were fed a standard fish meal diet to satiation daily. Fish near death from severe symptoms of infection during the observation period were removed and killed (by immersion in a lethal dose of MS222) before collection of fin tissue to minimize suffering. After the 3-week observation period, surviving fish were killed by immersion in a lethal dose of MS222 before sampling and disposal.

### Disease challenge and sample collection

Samples were collected from disease challenged fish and their parents in brood years 2008 and 2010 by staff at the Clear Springs Foods Inc. research facility in Buhl, Idaho. Healthy fish selected at random from the previous generation were artificially spawned to produce 2500 fertilized eggs from 130 families each during the course of 13 weeks. Fin tissue samples from each parent fish were collected at the time of spawning. The offspring were grown to ~1 g (62 days postfertilization) and 100 fish per family were selected randomly for disease challenge. Fifty fish from each family were infected by injection of 10 μL of a 0.2 Optical Density at 600nM wavelength (OD600) suspension of *F. psychrophilum* while the remaining fish were infected with IHNV by immersion into a volume of water 10x the total body weight of the fish in grams containing 10,000 plaque-forming units of IHNV per mL for 1 hr. After exposure, the fish were moved to 19-L tanks (50 fish/family/tank) and monitored for a period of 3 wk with mortality recorded daily. Each family/exposure group was kept in a separate tank. Fin tissue samples were collected from mortalities during the 3-wk monitoring period and survivor samples were taken at the conclusion of the challenge. The percentage of mortality for each family with each pathogen was recorded. This measure of disease resistance was determined to be accurate as mortality was rarely observed near the end of the observation period.

Mortality rates for each of the disease challenged families were examined and a broad range of mortality was targeted for each pathogen for inclusion in RAD sequencing. The mortality rates for CWD ranged from 4 to 96% and included 20 families. For IHNV family mortality range was 4–92% and also included 20 families. A total of 456 samples, which included parents and disease challenged offspring, were selected for RAD sequencing and comprised families from two brood years (2008 and 2010) with mortality ranging from low, high, and intermediate rates (Table 1). Each parent with roughly equal numbers of offspring mortalities (N = 5) and survivors (N = 5) were targeted for sequencing (Table 1). Note, mortality rates from families not selected for RAD sequencing ranged from 0–100%.

**Table 1:** Families evaluated for disease resistance and chosen for inclusion in association testing

Family ID	Disease Challenge	Year Class	Mortalities (Genotyped/Target)	Survivors (Genotyped/Target)	Mortality Rate
08-130	CWD	2008	2/2	3/5	0.04
10-125	CWD	2010	0/3	3/5	0.06
08-121	CWD	2008	2/4	5/5	0.08
08-092	CWD	2008	5/5	5/5	0.12
08-073	CWD	2008	5/5	4/5	0.13
10-060	CWD	2010	5/5	5/5	0.14
10-078	CWD	2010	3/5	5/5	0.16
08-105	CWD	2008	5/5	5/5	0.28
10-028	CWD	2010	5/5	5/5	0.36
10-127	CWD	2010	5/5	0/5	0.61
10-101	CWD	2010	4/5	4/5	0.64
08-025	CWD	2008	3/5	5/5	0.65
10-094	CWD	2010	3/5	3/5	0.69
08-129	CWD	2008	1/5	4/5	0.70
08-082	CWD	2008	5/5	5/5	0.79
10-119	CWD	2010	5/5	4/5	0.80
08-075	CWD	2008	4/5	5/5	0.86
10-076	CWD	2010	4/5	5/5	0.89
10-068	CWD	2010	4/5	5/5	0.91
08-108	CWD	2008	5/5	4/4	0.91
10-059	IHNV	2010	1/2	5/5	0.04
10-053	IHNV	2010	3/5	5/5	0.10
08-113	IHNV	2008	0/2	5/5	0.17
10-015	IHNV	2010	4/5	5/5	0.26
10-007	IHNV	2010	5/5	3/5	0.27
08-094	IHNV	2008	5/5	5/5	0.27
08-117	IHNV	2008	5/5	5/5	0.28
10-001	IHNV	2010	5/5	5/5	0.29
08-008	IHNV	2008	3/5	4/5	0.33
08-043	IHNV	2008	1/5	1/5	0.33
08-110	IHNV	2008	5/5	5/5	0.33
10-050	IHNV	2010	5/5	5/5	0.75
08-093	IHNV	2008	5/5	4/5	0.80
10-067	IHNV	2010	5/5	5/5	0.83
10-023	IHNV	2010	5/5	4/4	0.91
08-107	IHNV	2008	5/5	3/4	0.91
10-021	IHNV	2010	5/5	4/4	0.92
08-039	IHNV	2008	4/5	1/3	0.94
08-018	IHNV	2008	1/5	0/3	0.94
10-124	IHNV	2010	5/5	2/2	0.96

Families are ordered by disease challenge and mortality rate. CWD, cold water disease; IHNV, infectious hematopoietic necrosis virus.

### RAD library preparation

Fin tissue samples from individuals selected for RAD genotyping were used for DNA extraction using QIAGEN DNeasy 96 kits. Quantification of extract DNA was done using Invitrogen Quant-It pico green reagent and a PerkinElmer Victor V fluorimeter. Of the 456 samples chosen for inclusion in RAD library preparation, 27 had insufficient DNA concentration after extraction and quantitation. DNA extracts from the remaining 429 samples were normalized to 5 ng/μL and 500 ng of each sample was digested with *Sbf*1-HF restriction enzyme in NEBuffer 4 (New England Biolabs). Barcoded adapters were then ligated onto the cut ends of the restriction sites using T4 DNA ligase (New England Biolabs) and the samples were then pooled into libraries of 48 individuals each. The remaining steps of library preparation were carried out as described in Miller *et al.* (2012) and Hecht *et al.* (2012). The concentration of a 1:1000 dilution of each completed library was determined by quantitative polymerase chain reaction using Life Technologies PowerSYBR reagent and Kappa biosystems Illumina library DNA standards run on an Applied Biosystems 7900 instrument. Library concentration ranged from 6.5 to 71 nM after the addition of the P2 adapter and 15 cycles of PCR amplification. The concentration of each library was normalized to 5nM and sequenced on an Illumina HiSeq 2000 instrument.

### Genotyping pipeline

Raw sequencing data included 101 bases per read and averaged 151 million reads per library with a range of 143–245M (raw sequence data were submitted to the National Center for Biotechnology Information Sequence Read Archive database – SRA091643). The sequences were quality filtered, trimmed, and split into individual files based on barcode sequence using a custom perl script described in Miller *et al.* (2012). This process also strips the six base barcode and six base partial *Sbf1* site located on the 5′ end of each sequence while also trimming another 30 bases off of the lower quality 3′ end of each sequence. After quality filtering of the sequencing reads roughly 90% of the sequencing reads were retained. Of the reads passing quality filtering, roughly 70% began with a valid barcode and partial *Sbf1* sequence. The average number of reads per individual was 2.1M but with a standard deviation of 0.86M illustrating the read count variation between individuals.

Sequencing reads from three sets of parents (N = 6) were standardized to 2.4M reads each using the first 9.6M lines of each fastq file for alignment and SNP identification. The small number of individuals used for SNP discovery was chosen to capture most of the common SNPs and to reduce the amount of sporadic sequence errors interpreted as putative SNPs. These sequences were then combined and collapsed into unique sequences for alignment to one another using the program Novoalign (Novocraft, Selangor, Malaysia). The remaining steps for identifying SNPs within the RAD sequences and genotyping individuals were done according to the methods detailed in Hecht *et al.* (2012). In brief, a custom perl script was used to identify and output allele sequences for loci containing a single SNP site from the alignment data. Parameters for identification of SNPs within alignments of RAD sequences were set such that only those containing a single SNP which occurred more than 5 times within an alignment were collected. A total of 5647 putative SNP loci were identified within the sequencing data for these six individuals. Allele counts from each putative SNP locus were used to generate genotypes within all individuals in the study. A minimum read depth of 10 counts per locus was required in order for genotyping and genotypes were attempted in all individuals regardless of read count at this stage. Allele ratios were then used for determining genotypes for each RAD locus for each individual. Genotypes were scored using the following ratios [Allele 1 Homozygote > 7/1 > Heterozygote > 1/7 > Allele 2 Homozygote].

### Genotype filtering

Genotype data were refined by eliminating individuals and SNP loci with more than 20% missing genotypes. Observed and expected heterozygosity (H_obs_ and H_exp_) was calculated for each locus and differences in these values of greater than 0.4 were excluded from the data set. Further, Hardy-Weinberg equilibrium was tested for each locus, and any loci deviating significantly from equilibrium were removed to reduce the inclusion of paralogous sequence variants. After filtering, a final data set containing 384 individuals genotyped at 4661 SNP loci were used to generate an input file for the program TASSEL (Bradbury *et al.* 2007) to test for association between genotype and resistance to either CWD or IHNV. Of the 384 individuals genotyped, 159 were challenged with CWD, and 153 were challenged for IHNV (Table 1). The remaining 72 fish were parents of the disease challenged fish but had no associated phenotype because they were not directly challenged. The parent samples were not included in GWAS analysis.

### GWAS analysis

The TASSEL program uses either a general linear model (GLM) which may include covariates or a mixed linear model (MLM), which includes a kinship matrix in addition to any covariates to determine association between traits and phenotype. In our case, we described disease resistance as a simple binary trait where mortalities were given a value of 1 and survivors a value of 2. Several types of covariates were generated for inclusion in both the GLM and MLM analysis including STRUCTURE Q-coordinates (Pritchard and Wen 2003), factorial coordinates (GENETIX: Belkhir *et al.* 1996), and principal components values (GENALEX 6: Peakall and Smouse 2006). Covariate data were generated using 1300 SNP loci with 95% genotype frequency or greater and 20% or greater minor allele frequency. A kinship matrix was generated using the EMMA algorithm in the program GAPIT (Lipka *et al.* 2012) for inclusion in the MLM analysis using the same loci used for the covariate data. We determined which and how many covariates for our final data set using various iterations of the GLM and MLM analysis in TASSEL and subsequent evaluation of QQ-plots. The final analysis used six principal components as covariates along with the kinship matrix in the MLM. Statistically significant loci were identified by applying a BY-FDR correction for multiple tests (false discovery rate; Benjamini and Yekutieli 2001).

Following the identification of SNP loci associated with disease resistance, the TASSEL program was used to generate genetically evaluated breeding values (GEBVs) for each of the fish in the study. This analysis uses best linear unbiased predictor values for each locus to determine GEBV. For comparison of GEBV data from IHNV and CWD, each value of GEBV was converted to a number between 0 and 1 representing the upper and lower bounds of the distribution.

### Mapping of RAD markers

For mapping significantly associated loci to linkage groups (LGs), we used RAD sequences generated from Miller *et al.* (2012) to match identical loci in our data that used the same restriction enzyme (*Sbf1*). We then filtered the matching loci to those containing map positions (*N* = 721). LGs for significant loci with direct matches to mapped loci from Miller *et al.* (2012) were recorded (N = 4). Genotypes for the mapped loci and the significantly associated loci were used for pairwise LD tests using GENEPOP (Raymond and Rousset 1995). All samples were treated as a single population for the LD test using the standard settings (dememorization 10,000; batches 100; iterations per batch 5000). Output data were filtered for pairwise comparisons containing significantly associated loci, and perl was used to add LGs and map positions to the data. A Bonferroni correction (α = 0.01) for significance was used to filter *P*-values (*P* < 0.00001339) for each pairwise comparison. LG was determined by examination of the map position of all loci determined to be in statistically significant LD. In some cases the LG was ambiguous, and LG was recorded as not determined (Table 2). LG names are reported according to both Miller *et al.* (2012) and Palti *et al.* (2011). LG number and chromosome number were resolved by Phillips *et al.* (2006) and are reported as such in the genetic map of Palti *et al.* (2011. This map also was used to determine proper chromosome numbers in previous QTL studies by matching common microsatellite markers.

**Table 2:** RAD markers showing significant association with disease resistance

Marker	Association	*P* Value	*BLUP*	LG	Marker Sequence
R45138	CWD	1.49E-05	1.78E-01	WS08/Omy16*^a^*	TGGTCGCAAGGGGAAACATAGCCGCCATAGGCATCCTAAGCCTTTTAGGGC[T/A]GCAAAAC
R46743	CWD	5.31E-04	9.13E-03	WS08/Omy16*^a^*	TTCATTCTCAACAACATCCAT[G/A]GAGTATGACCTTACCTCACAGAGGAAGCGGCGCAGGT
R26956	CWD	6.33E-04	1.23E-01	WS23/Omy17	GGAGGAAGAGAGGATGTGGGGGAGGAAGAGAGGATGTGGG[G/A]GAGGTTGGAAACATGATT
R46637	CWD	1.77E-03	−5.24E-02	WS05/Omy8*^b^*	CCTTGGTAGTGGGCCGTGTCGGTGGCACTG[T/C]ATTATCCTCAAAGCTGGTAAAGAAGGTG
R08795	CWD	1.91E-03	1.42E-01	Omy29+	CCACTTTCTGTCGCTCTCTTTGTCTCAGTCATTCCCTTTCTT[G/T]TGATTTCTTTCCCTTC
R13883	CWD	2.79E-03	−1.43E-01	WS13/Omy06	TGCAGTTCTGAAGGTTGATTCAGCAGTATTTGGGGCATCGAAGTGATGGGACAT[A/G]TGAC
R49259	CWD	3.06E-03	1.49E-01	NA	AAATCACGCACAGAACAAGC[A/T]GTACGGCTGGTGGTATTGTCATGCTTGAAGGTCATGTC
R34531	CWD	3.29E-03	−5.92E-02	WS13/Omy06*^a^*^,^*^c^*	TGATGCCCATGGCCTTGATCTTGGCCGA[T/C]TCGCTGCCCAGCTCGTTCAACTGCTGTTTA
R40622	CWD	3.52E-03	7.22E-02	WS01/Omy04	AGAGTAGA[C/T]CTCCAGGAACAGGTTAGGCCACCACTGACAAAGAACATCCAGCATTAGAG
R46597	CWD	3.99E-03	7.56E-02	WS23/Omy17	[C/T]GGTTTAGGACCTTTGTAATGACTTTCATTTAGCTAGCGCAAGTATTTGGTTCTGGGTT
R20199	CWD	4.03E-03	1.14E-01	WS01/Omy04*^a^*	AAGGGTAACACATTTCCTGGGTTATTCAACTGGCCCCTCCGTCG[C/T]GACGTCCTCTGAAT
R53165	CWD	4.45E-03	−2.64E-02	WS04/Omy9*^b^*	TCTGTGACATCAACATGCTGTTTTCCTCAGTAGGGTCATGCAAAAACAAATG[T/G]CACCTT
R21407	IHNV	5.98E-05	5.24E-02	WS13/Omy06*^a^*	GAGTGAGAATGGAGGACAAGAAGGTAGATG[G/C]GGTAAGGCCATGGCCAGTCCCAACCACC
**R14353**	IHNV	9.52E-05	−1.33E-01	WS13/Omy06^a^	AGTACATAACATGAC[C/T]GTACATATTTAATATGCTATTCAAGTGTTTTAAGACACCAATA
R24813	IHNV	8.95E-04	7.86E-02	WS13/Omy06*^a^*^,^*^c^*	TTTGACTCCTGATGAATGCCCCGTCTGCGGAATCTCTCTTGCTCGCTCTCTC[A/G]GCAATC
R53135	IHNV	9.53E-04	−1.23E-01	NA	CACATTGCCTGGTGGCGTTTATTAATGTTTACATACTGTACCGTTT[A/C]TTTTTTTTTAAA
R03138	IHNV	1.01E-03	9.38E-03	WS13/Omy06*^a^*^,^*^c^*^,^*^d^*	TGCCCGGCCCGCCACAAGGAGTCGCTTGA[A/G]CGAGTAAAGTCCCCCTGGTTGTGATACAG
R39227	IHNV	1.45E-03	1.49E-01	WS07/Omy11*^d^*	C[C/A]TCCAGCTGCCTGCCTCACAGGCCAAATATGCTATTTAGAAAATTGGGATATAAGAGA
R53350	IHNV	1.63E-03	−1.53E-02	WS13/Omy06*^a^*	CCAAACTCTCCCCTAACCCGGACGACGCTGGGAAAATTGTA[C/T]GCCGCCCTATGGGTCTC
R52586	IHNV	1.66E-03	5.55E-02	WS28/Omy26	TAGACTGTAC[T/C]AAGAGCTGAATCACAACTCCTGTATGGATAACACCACACTGGTCAAGT
R46528	IHNV	1.78E-03	−6.56E-02	WS24/Omy25	AGTGAGTCACTG[C/A]TTTGATGTTTGCAGAGCACGACAGAGTGTTGTGCAGGGTCATTGCA
R48860	IHNV	2.44E-03	1.37E-01	WS14/Omy10	CGCTCGCCC[T/C]GCCACAAGGAGTCGTTAGAACGCGATGAGCCAAGTAAAGCCCCCCAAGG
R33079	IHNV	2.46E-03	−1.16E-02	WS13/Omy06*^a^*^,^*^d^*	CCATGGGCAACCTCACCCCTCTCCATAC[C/G]CCTACTCCTCCACAGTCCCCTCAGAGCTCA
**R52674**	IHNV	2.54E-03	−6.30E-02	WS13/Omy06*^a^*	GGACCCACCAATCCTGAAGAAGTTAAAGACTCCAGCCATTCAAGC[C/A]ATAGACTGGCTGG
R19689	IHNV	3.29E-03	1.14E-01	WS03/Omy05	ATCATCTGAGACCAGCCACCCAGACAGCTGTGGGTTTGTGGGTTTGCACAACCAAATA[A/T]
R04597	IHNV	4.07E-03	−7.38E-02	WS03/Omy05*^a^*	CTCATCCTGACATGACCCTGCAGCTTGACAATGTCACCAGCCATACT[G/A]CTCGTTCTGAG
R14972	IHNV	4.19E-03	1.80E-02	WS13/Omy06*^a^*	CTACCACTCTTTCCCCAAGTAAGTA[T/C]CCGGGTGAATGGTTAAACAACTAGCCTAAACAG
R18850	IHNV	4.19E-03	−8.30E-02	NA	TCTGGTGTCAGAATGCGCA[C/A]GGAGCTCCTCAGTGGATTACTTCTGGTACAGAGAGACAC
R45284	IHNV	4.37E-03	7.09E-02	WS29/Omy13*^a^*	CTACTATTAAAGTATCACTGCTTTGGTGTGTAATTCCAAT[G/A]CCCCTTTATCTTTTAAGT
R05136	IHNV	4.87E-03	−2.22E-02	WS14/Omy10*^a^*^,^*^c^*	ACGGG[A/T]ATCACAATAGGGAGGAGGATGTCTTCCCTGTAATGCACAGTGTTAAGATTGCC
R52799	IHNV	5.16E-03	−1.76E-01	NA	GCTACAGACAGAC[T/G]ATCACTCTATCATGGTAGGATGGATGGAGGGAGGGAGCACATCAC

Linkage group (LG) names are as reported by Miller *et al.* (2011)/Palti *et al.* (2011). The two bold and underlined loci align to the same contig in the *O. mykiss* draft genome. Marker sequences follow the SbfI recognition sequence “CCTGCAGG.” CWD, cold water disease; IHNV, infectious hematopoietic necrosis virus; RAD, restriction site−associated sequencing; BLUP, best linear unbiased predictor.

a LG confirmed by alignment to Berthelot *et al.* (2014) *O. mykiss* genome.

b Directly matches mapped RAD locus from Miller *et al.* (2011).

c LG assignment supported by Berthelot *et al.* (2014) *O. mykiss* genomoe alignment only.

d LG assignment confirmed by alignment to same draft genome contig as previously mapped RAD marker.

The program BOWTIE (Langmead *et al.* 2009) was used to search an *O. mykiss* draft genome assembly (available at: http://www.animalgenome.org/repository/aquaculture/) for significantly associated RAD locus sequences. Search parameters allowed for up to two mismatches per 59 base RAD sequence and reported all contig matches. These data were used to validate LGs assigned to significant RAD markers by co-occurrence of disease resistance associated RAD markers and mapped RAD loci within draft genome contigs.

Additional mapping of RAD loci was done by using the program BOWTIE (Langmead *et al.* 2009) to identify the position of each locus within the recently released rainbow trout genome (Berthelot *et al.* 2014). Search parameters allowed for up to two mismatches per 59 base RAD sequence and reported the best match for each locus. The base position of each aligned locus was used to order the markers by chromosome and position (Figure 2). These data also were used to confirm the proper assignment of significantly associated loci to LGs by LD analysis (Table 2).

### Power analysis

A simple power analysis was conducted using the program CaTS (Skol *et al.* 2006) to estimate our power to detect genetic association. Input parameters for the program were manipulated to reflect the sample size, average allele frequency of our marker set, and significance level for detection. Using the additive model, we set the disease prevalence to the average family mortality rates observed among the families challenged for each pathogen (0.49 for CWD and 0.53 for IHNV) and genotype relative risk was set to 1.3. Because the program will not accept sample sizes smaller than 100 individual cases/controls, a power curve was generated using sample sizes from 500 to 100. The power at the sample sizes used in this study (CWD: 75 cases/84 controls; IHNV: 77 cases/76 controls) was extrapolated based on these curves (Supplemental Data 2). However, because the average genotyping rate was 95.1%, the effective sample sizes varied among loci and averaged approximately 159 samples.

### Identification of genes near resistance−associated RAD loci

Because the Berthelot *et al.* (2014) rainbow trout genome included gene annotations, it was used to identify genes nearby significantly associated RAD loci. The genome location of each locus was determined by alignment of each RAD tag sequence to the genome using BOWTIE and a custom Perl script was used to collect any CDS within 50K bases. Since CDS within the genome are not identified, each sequence was used in a BLAST-X search using the National Center for Biotechnology Information database to determine gene homology.

## Results

**Figure 1:**
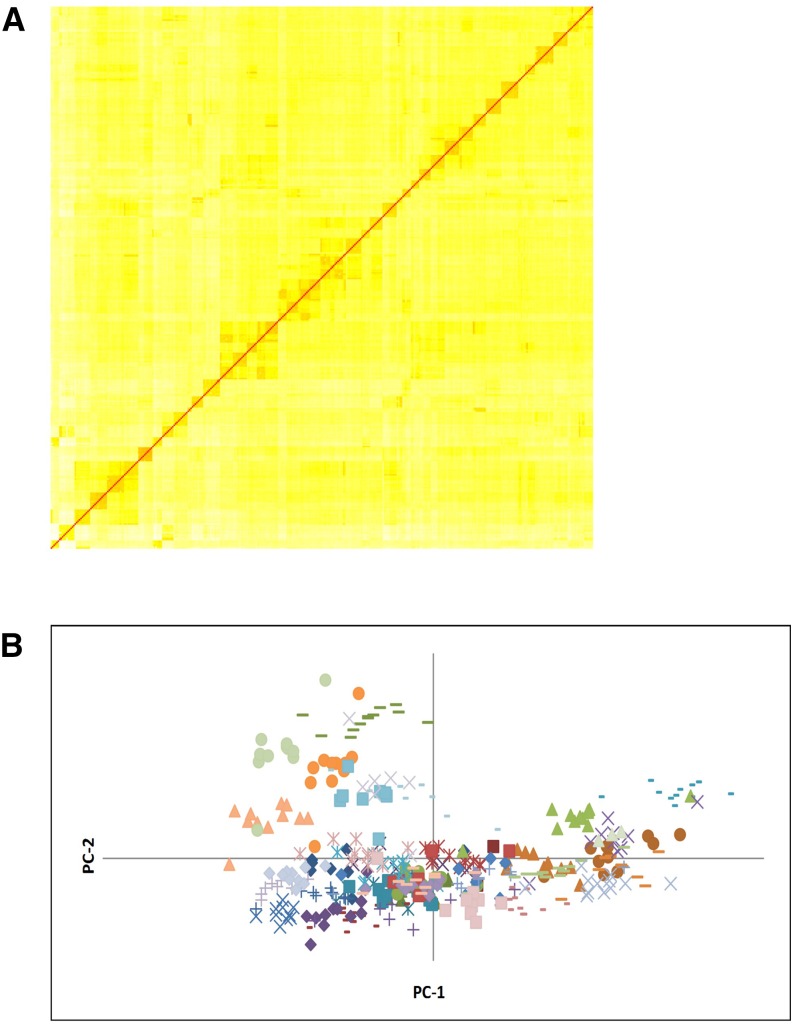
(A) Heat map of pairwise kinship among individuals included in the study. Red squares indicate an individual’s genetic relatedness to itself and orange blocks indicate genetically reconstructed family groups. (B) Principal coordinates plot of all individuals. Family groups are indicated by marker color/shape. PC, principal coordinate.

This study was designed to reduce family effects by including roughly equal numbers of mortalities and survivors from each family cross. After filtering however, some family groups contained uneven numbers of mortalities and survivors (Table 1). Also, because the data were collected from a captive hatchery strain, it was possible that more cryptic relatedness between families could yield false positive associations. To account for kinship in the GWAS model, a subset of 1300 SNP genotypes were used to produce a kinship matrix that accurately reconstructed all 40 families included in the analysis and also revealed other relatedness patterns among family groups (Figure 1A). The same subset of SNP genotypes also were used to generate principal components values using the program GenAlEx. This analysis also illustrated the ability of the genotype data to accurately cluster individuals into known families (Figure 1B).

**Figure 2:**
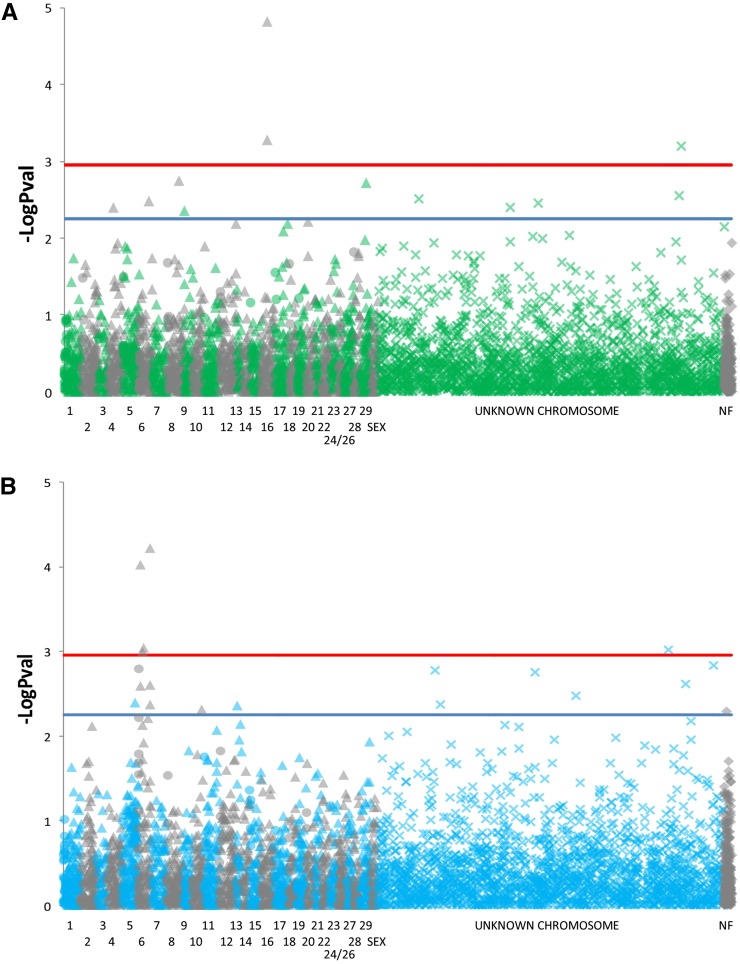
Plots of significance values for each marker associated with resistance to each disease. (A) Bacterial cold water disease (CWD). (B) Infectious hematopoietic necrosis virus (IHNV). Horizontal lines in each figure represent increasingly stringent critical values where blue line is BY-FDR α = 0.05, and the red line is BY-FDR α = 0.01. Markers are ordered according to alignment to Berthelot *et al.* (2014) genome position. Circles indicate a match to the properly scaffolded portion of each chromosome while triangles indicate a match to the unordered portion of the chromosome. An “x” indicates a match to the unknown chromosome and gray diamonds indicate that the marker was not found within the genome assembly.

Association analysis using the program TASSEL was conducted using several covariates in both the general and mixed linear models. Most of the significantly associated loci remained largely the same regardless of the number or type of covariates used. Q-values were quickly eliminated as a viable option for this study due to the genetic homogeneity of the hatchery strain and inability to identify the number of distinct founding populations. Factorial coordinates and principal coordinates used as covariates produced nearly identical results. However, since both principal coordinates and factorial coordinates provided similar data, PCs were chosen as they are the most commonly used of the two in association mapping. After examination of QQ plots (File S1) using three and six PCs, a combination of six PCs as covariates was chosen along with the kinship matrix in the MLM analysis. Statistically significant loci were identified using BY-FDR corrections for multiple tests (false discovery rate; Benjamini and Yekutieli 2001), which produced a significance cutoff of *P* < 5.54 × 10^−3^ at α = 0.05. In total there were 31 loci significantly associated with either IHNV or CWD survival identified (Table 2), with 12 markers that were highly associated with resistance to CWD and 19 markers associated with resistance to IHNV (Figure 2).

**Figure 3:**
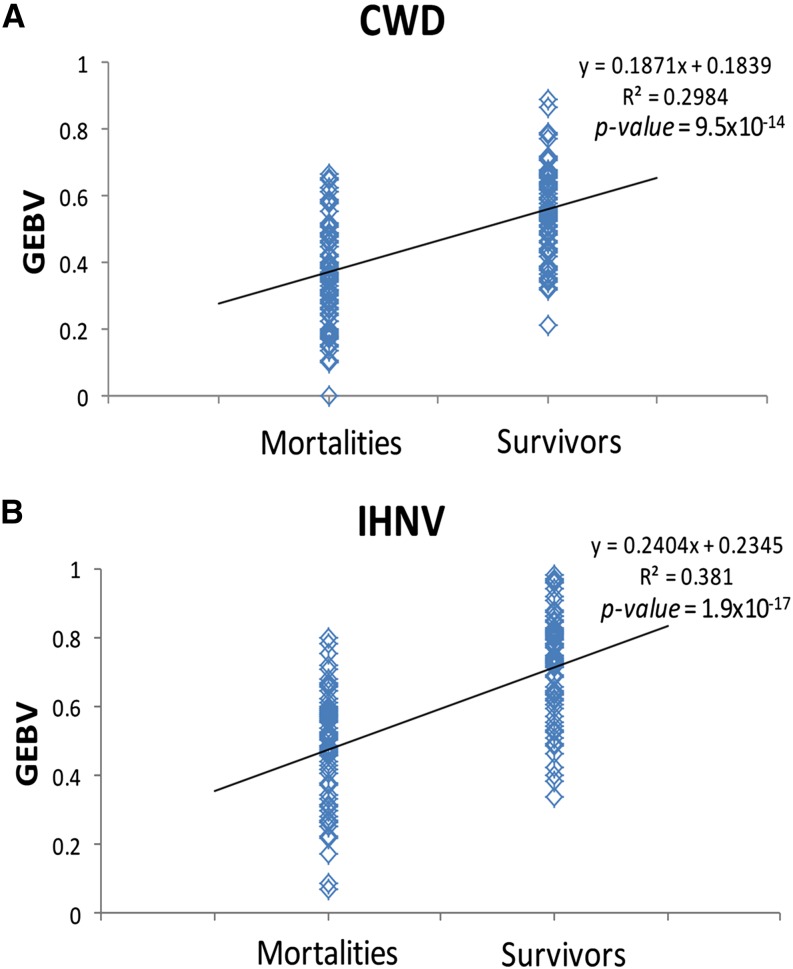
Distribution of genetically evaluated breeding values (GEBVs) among disease challenged fish using significantly associated markers for (A) cold water disease (CWD) and (B) infectious hematopoietic necrosis virus (IHNV).

To assess the value of these markers for prediction of survival to disease exposure we generated GEBVs. We calculated GEBVs for all the individuals in our study by using markers that were significantly associated with resistance to each disease. The distribution of GEBVs between known mortalities and survivors (Figure 3) was examined by analysis of variance and found to be highly significant between the survivor and mortality groups for both diseases (IHNV: *P* = 1.9 × 10^−17^; CWD: *P* = 9.5 × 10^−14^).

Disease resistance associated loci were mapped to LGs using LD analysis by matching RAD loci from a previously generated genetic map (Miller *et al.* 2012). Using a custom perl script to identify common allele sequences, we were able to identify 721 mapped RAD loci contained in our genotype data. A filtered set of genotype data that contained only mapped and significantly associated RAD loci was then used for LD analysis using the program GENEPOP. LG was determined on a per locus basis considering mapped loci with significant Bonferroni-corrected *P*-values (α = 0.01; *P* < 1.34 × 10^−5^). For most of the disease resistance associated loci (25 of 31) a confident LG assignment could be made when significant LD was observed with multiple mapped RAD loci from the same LG (Table 2). However, there were six loci with statistically significant LD with roughly equal numbers of mapped loci representing several LGs and therefore could not be determined. A separate approach for assignment of these markers to LGs using BOWTIE to examine co-occurrence within the same draft genome contig with mapped markers further validated the LD analysis for three of the RAD markers. Unfortunately there were too many individual contigs within the draft *O. mykiss* genome and too few mapped markers making sequence matches within the same contig rare (Table 2). However, similar analysis using the genome assembly of Berthelot *et al.* (2014) was able to confirm 15 of the assignments made using LD analysis and determine LG for another three loci (Table 2). Of the disease resistance loci we could map, several LGs were observed multiple times. For instance, chromosome 6 was represented in eight of the 19 IHNV-associated RAD loci and two of the 12 CWD-associated loci, indicating that this genomic region plays an important role in disease resistance (Figure 2 and Table 2).

Power analysis using the program CaTS indicated 17% average chance of detection of association in the CWD GWAS and 14% in the IHNV GWAS given our sample size and the average allele frequency of our markers using the additive model. This estimate also assumes a genotype relative risk of 1.3 and a disease prevalence set to our average observed mortality rates among the test families (0.49 for CWD and 0.53 for IHNV). The significance level for detection of association was set to our Bonferroni corrected p-value of 5.54 × 10^−3^.

The program BOWTIE was used to locate the disease resistance associated RAD loci within the Berthelot *et al.* (2014) *O. mykiss* genome and to identify genes within 50K bases. Of the 31 associated RAD loci, 19 were located within a numbered chromosome assembly, another 12 were located within the “unknown” chromosome, and a single marker (R52799) was not located within the genome. This analysis identified 155 total CDS located near 26 of the significantly associated RAD loci. Sequences from each of these CDS (File S3) were used in a blast-x search to determine gene homology. Results are summarized in File S4.

## Discussion

Association analysis uncovered 31 SNP loci significantly associated with resistance to either IHNV or CWD in rainbow trout. Moreover, when these associated loci were analyzed collectively, they demonstrate potential to predict an individual’s resistance to disease. However, unlike QTL analysis which generally is performed using the offspring of a known F2 cross, association studies simply use populations of “affected” and “unaffected” individuals that are not necessarily related. Therefore, association studies require a closer proximity between the trait gene and a linked genetic marker due to linkage decay. Because rainbow trout have a genome size of about 3B bases and used a restriction enzyme with an eight-base recognition sequence, our RAD loci were expected to average 65K bases apart. However, because the RAD sequences also must contain a single biallelic SNP site, true marker density averages only one marker every 650K bases. It is therefore likely that there are genomic regions associated with disease resistance that were not detected due to lack of LD with a nearby marker. A previous study in rainbow trout indicated that significant syntenic LD (r^2^ > 0.25) deteriorated at genetic distances over 2 cM, yielding a sex averaged estimate of 1500 markers needed for 1x genome coverage (Rexroad and Vallejo 2009). This estimate indicates an approximate 3x genome coverage using our set of 4661 RAD markers. However, estimates of linkage decay in other organisms using greater marker densities and locus pairs with known physical distances indicate that the numbers of markers needed for full genome coverage may be roughly an order of magnitude greater (Marques *et al.* 2008; Badke *et al.* 2012). Combining genome coverage information with our power analysis results gives a range of detection probabilities between about 5.7% and 43%. This range is incredibly broad due to the uncertainty surrounding the estimates of linkage decay but indicate that our power to detect loci associated with disease resistance is significant however low. For future studies, the numbers of affected/unaffected individuals should be increased at least 3 fold in order to improve the likelihood of detection of associated markers. Increased marker density would also allow for more thorough coverage of the genome to test for associations.

Selective breeding of this strain of rainbow trout was first implemented for resistance to CWD in 2001 and IHNV in 2000. Since broodstock fish are bred as 2-yr-olds, three to four generations of selective breeding had taken place before samples were taken for this project in 2008 and 2010. Under conditions of strong selection, alleles of large effect could have been pushed to near fixation in the interval between starting selective breeding and collection of samples. If this is the case, then genomic regions with the strongest contribution to disease resistance would not have been detected in the current study. However, significant variation in family performance when exposed to each pathogen was still observed in these years and care was taken to select samples from families with a wide range of mortality rates. Moreover, broodstock fish were selected based on family performance in a combination of survival in third-use water and growth, as well as resistance to IHNV and CWD. We suspect this combinatorial approach softens the selective pressure for disease resistance allowing the detection of associated genetic markers even after several generations.

False-positive associations resulting from enriched traits within certain populations and families often confound genetic association studies (Pritchard *et al.* 2000). For this reason great care is taken to discern true genetic associations from those resulting simply from genetic similarity between affected individuals. In this study this concern was addressed using three approaches. First, roughly equal numbers of mortalities and survivors were targeted from each family for RAD sequencing. By doing this both the control group and test groups had matching genetic backgrounds. Second, a kinship matrix was generated for use in the MLM analysis, which allowed for the subtraction of relatedness from the best fit linear regression to discern true associations from those resulting from family effects. Finally, PCA coordinates were used as a measure of the genetic background of each individual fish in the study. Similar to the kinship values, these are subtracted from the linear model to test whether the trait is more closely correlated with genotype or genetic background. Using these filters, a relatively small number of associated markers were identified (N = 31; IHNV = 19; CWD = 12) representing only a few LGs. Of the 10 implicated LGs, five have been previously reported to contain immune genes (Phillips *et al.* 2006, 2013) and significant associations to three of six chromosomes were reported previously to contain QTL for IHNV resistance in rainbow trout outcrosses (Rodriguez *et al.* 2004; Barroso *et al.* 2008). However, these studies did not implicate our most commonly associated chromosome (Chr6) as containing QTL for IHNV resistance but chromosome 6 is known to contain the immune gene *Il1β* (Phillips *et al.* 2013), which is located within the “chrUn_6” sequence of the genome of Berthelot *et al.* (2014) within approximately 2M bases of one of the significantly associated markers (R24813). However, the contigs within this genome assembly are unordered so although these loci are known to occur on chromosome 6 their actual proximities remain unknown.

For this study we used LD analysis with previously mapped loci to assign most of our significantly associated RAD loci to specific chromosomes. This strategy of using LD analysis in GENEPOP was approached with caution because using a large number of related individuals may give false-positive linkage results. However, the results obtained from the analysis were mostly unambiguous, and many loci could be assigned to a LG with confidence. Later analysis using genome alignments to examine co-occurrence of significant markers and mapped markers within both available draft genomes provided further validation of the results obtained using LD analysis (Table 2). Although the draft genomes provide a much needed resource for genetic studies in *O. mykiss*, they are not sufficient to determine accurate genome positions for genetic markers. We were able to locate approximately 92% of our RAD markers within these genome assemblies. However, these genomes are composed largely of unordered contigs, and although the Berthelot *et al.* (2014) genome has been assembled into chromosome sequences, the majority of contigs within each sequence is in undetermined order and approximately half of the genome sequence is not assigned to any chromosome. Further development of this resource using either greater density genetic maps or additional scaffolding to reduce the number of contigs and improve the chromosome scaffolds will ultimately make genome mapping more precise.

To illustrate the genetic effect of the significant markers collectively, we used analysis of variance of the GEBV among the mortalities and survivors for each pathogen. The result demonstrates significant differences in GEBV between survivors and mortalities for both tests. However, this test is not a robust one of predictive ability because the same individuals were used for GWAS and GEBV. Validation of the effect using individuals not included in the GWAS is necessary in order to confirm the predictive ability of these markers. Although this test does demonstrate the predictive power of the markers in determining disease resistance provided the individuals used for GWAS are representative of the population as a whole and the markers identified as significant are not false positives.

This study provides an example of how RAD sequencing can be used to generate thousands of SNP markers and conduct GWAS in species with limited genomic resources, although ideally future studies should use more samples with greater marker densities. The ability to identify genetic markers associated with physical traits makes applied genetic techniques such as MAS possible within these species as well. In this study, RAD sequencing was used to identify and genotype 4661 SNP markers segregating within a hatchery population of rainbow trout. Further, trait data were utilized to isolate 31 markers associated with survival following exposure to IHNV or CWD. Using GEBVs generated using genotypes at resistance associate loci only; this work also demonstrates the ability to use these markers to predict phenotype. Finally, 27 of our 31 resistance-associated loci were successfully mapped to LGs by matching RAD loci to a set of previously mapped loci by LD analysis and by their identification within a genome assembly for this species (Berthelot *et al.* 2014). These markers offer a foundation for further study of the underlying genomic regions related to resistance to IHNV and CWD in rainbow trout and incorporation of marker assisted selection to aquaculture programs for this species.

## Supplementary Material

Supporting Information
