# Supplementary material for: Association Mapping of Disease Resistance Traits in Rainbow Trout Using Restriction Site Associated DNA Sequencing
Source: G3 (Bethesda). 2014 Oct 28;4(12):2473–81. doi: 10.1534/g3.114.014621 (PMC4267942; doi:10.1534/g3.114.014621)
Supplement: Supporting Information [file supp_4_12_2473__index.html]

Association Mapping of Disease Resistance Traits in Rainbow Trout Using Restriction Site Associated DNA Sequencing — Supporting Information 

# Association Mapping of Disease Resistance Traits in Rainbow Trout Using Restriction Site Associated DNA Sequencing

## Supporting Information for Campbell *et al.*, 2014

**Files in this Data Supplement:**

- File S1 - Images of QQ plots for each of the GWAS tests. (PDF, 55 KB)
- File S2 - Power curves for each of the power analyses performed. (.xlsx, 26 KB)
- File S3 - Fasta formatted sequences of CDS within 50K bases of associated RAD loci. (FASTA, 204 KB)
- File S4 - Top blastx hits for each of the CDS sequences found within 50K bases of associated RAD loci. (.xlsx, 16 KB)
