# Supplementary figures and images for: Association Mapping of Disease Resistance Traits in Rainbow Trout Using Restriction Site Associated DNA Sequencing
Source: G3 (Bethesda). 2014 Oct 28;4(12):2473–81. doi: 10.1534/g3.114.014621 (PMC4267942; doi:10.1534/g3.114.014621)

**Expected -Log(P-Value) vs. -Log(P-Value)**

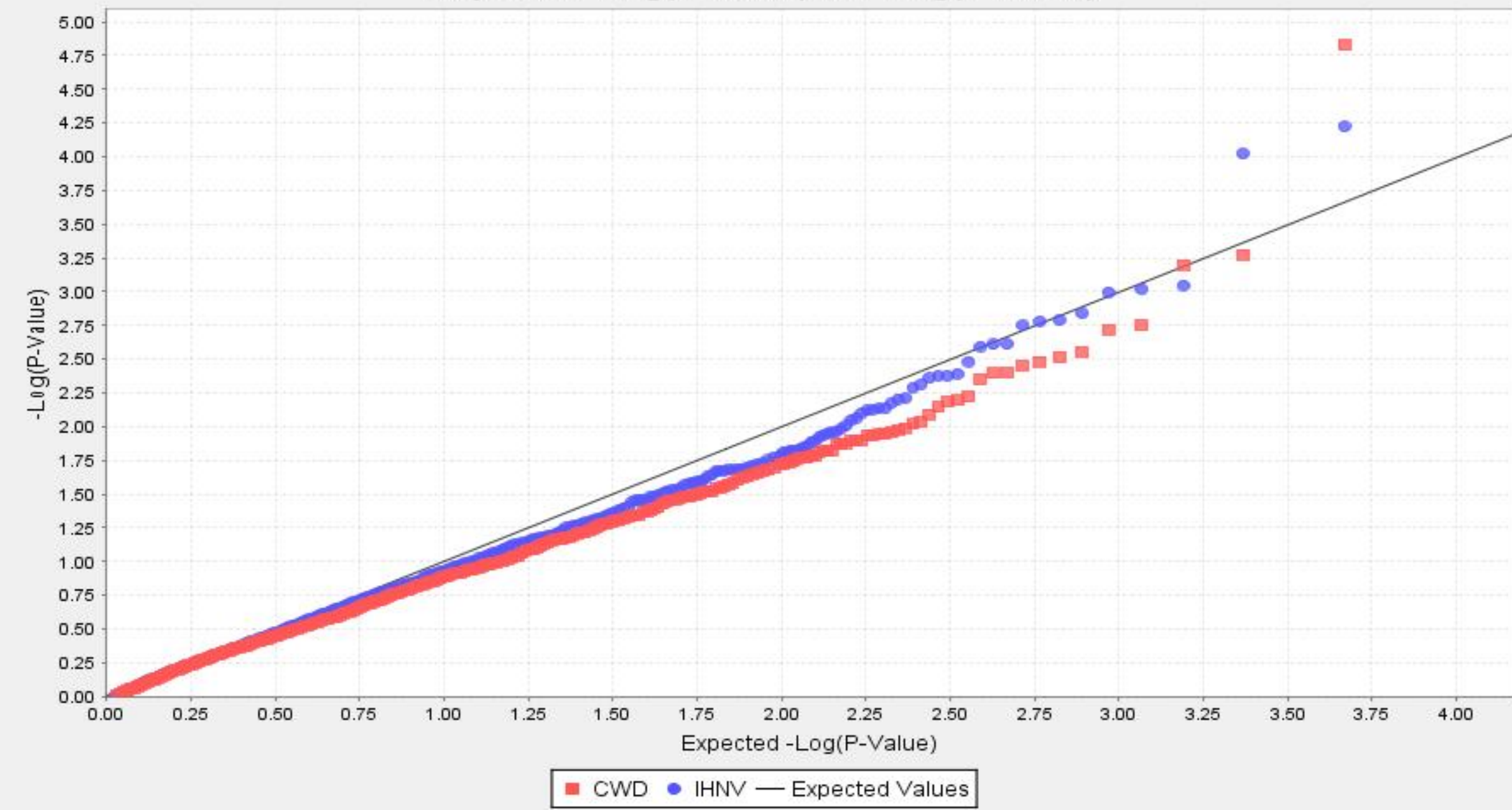

Supplement: Supporting Information [file supp_g3.114.014621_FileS1.pdf]
